# The association of cognitive function and its changes with all-cause mortality among community-dwelling older adults

**DOI:** 10.3389/fnagi.2024.1419235

**Published:** 2024-06-12

**Authors:** Shangjie Li, Xiuping He, Liang Wu, Xinming Tang, Yijiang Ouyang, Wenyuan Jing, Ya Yang, Jiacheng Yang, Kechun Che, Congcong Pan, Xiaoting Chen, Xiaoxia Zhang, Xueting Zheng, Jiahao Xu, Shaobin Liao, Mingjuan Yin, Jindong Ni

**Affiliations:** ^1^Shunde Women and Children’s Hospital, Maternal and Child Research Institute, Guangdong Medical University, Foshan, China; ^2^Precision Key Laboratory of Public Health, School of Public Health, Guangdong Medical University, Dongguan, China; ^3^Community Health Center of Dongguan Songshan Lake (Public Health Office), Dongguan, China

**Keywords:** older adults, cognitive impairment, change, all-cause mortality, cohort study

## Abstract

**Background:**

The association of cognitive function, its changes, and all-cause mortality has not reached a consensus, and the independence of the association between changes in cognitive function and mortality remains unclear. The purpose of this study was to evaluate the longitudinal association between baseline cognitive function and cognitive changes over 1 year with subsequent all-cause mortality among the older adults aged 60 and above.

**Methods:**

A prospective cohort study utilizing the Community Older Adults Health Survey data. Initiated in 2018, the study annually assessed all individuals aged 60+ in Dalang Town, Dongguan City. Cognitive function was assessed using the Chinese version of the Mini-Mental State Examination (MMSE). A total of 6,042 older adults individuals were included, and multivariate Cox proportional hazard models were used to examine cognitive function’s impact on mortality.

**Results:**

Participants’ median age was 70 years, with 39% men. Over a median 3.08-year follow-up, 525 died. Mortality risk increased by 6% per MMSE score decrease (adjusted *HR* = 1.06, 95%*CI*: 1.05–1.08). Compared to those with normal cognitive function at baseline, participants with mild cognitive impairment and moderate to severe cognitive impairment had significantly higher mortality risks (adjusted *HR* = 1.40, 95%*CI*: 1.07–1.82; *HR* = 2.49, 95%*CI*: 1.91–3.24, respectively). The risk of death was 5% higher for each one-point per year decrease in cognitive function change rate (*HR* = 1.05, 95%CI: 1.02–1.08). Compared with participants with stable cognitive function, those with rapid cognitive decline had a 79% increased risk of death (adjusted *HR* = 1.79, 95% *CI*: 1.11–2.87), with baseline cognitive function influencing this relationship significantly (*P* for interaction = 0.002).

**Conclusion:**

Baseline cognitive impairment and rapid cognitive decline are associated with higher all-cause mortality risks in Chinese older adults. Baseline function influences the mortality impact of cognitive changes.

## 1 Introduction

The World Health Organization estimates that the global population aged 60 and over will increase to 1.4 billion by 2030 (United Nations, 2023). As the global population undergoes aging, the associated issues are becoming more pronounced. This includes cognitive decline, which, in severe cases, can develop into cognitive impairment, and subsequently dementia. Cognitive impairment generally refers to varying degrees of cognitive function decline caused by several factors (including memory, computation, temporal and spatial orientation, structural ability, executive ability, language understanding, expression and application, etc.,) (Ni et al., 2022). It is now established that cognitive impairment is associated with various adverse outcomes such as falls, prolonged hospitalization and readmission (Buslovich and Kennedy, 2012; Callahan et al., 2015; Ma et al., 2021b). Dementia ranks as the seventh leading cause of death and it is one of the primary reasons for dependency and loss of autonomy among older adults worldwide (World Health Organization, 2023). However, the association between cognitive function and its changes with all-cause mortality lack adequate evidence from existing research.

Some studies have indicated that cognitive impairment in older adults is associated with an increased risk of all-cause mortality (Duan et al., 2020; Su et al., 2021; Diniz et al., 2022), but these studies only considered cognitive function at a single point in time. Research have shown that changes in cognitive function affect the risk of mortality in older adults. Recent cognitive decline or rapid cognitive decline has the higher risk of mortality while compared to stable (Bassuk et al., 2000; Hu et al., 2019). Furthermore, the joint progression of cognitive decline and physical frailty is associated with a higher mortality risk in older adults (Chen et al., 2023). Nonetheless, some studies report that, after adjusting for baseline cognitive function, a decline in cognitive ability does not add an extra mortality risk (Bruce et al., 1995). Research indicates that older adults have similar mortality rates when their cognitive decline reaches a higher degree, regardless of their baseline cognitive function, negating any life expectancy benefits of better baseline cognitive function (Tsui et al., 2022). Moreover, the dose-response relationships between baseline cognitive function, its changes, and mortality have not been accurately evaluated. In China, research on the association between cognitive function changes and mortality among community-dwelling older adults is lacking. Additionally, with significant changes in lifestyle, urbanization, and living environments in China over the past decade, the cognitive function of older adults and its changes may have also shifted, potentially affecting the association with mortality (Jia et al., 2020). This indicates the necessity for updated reports.

We are yet to reach a consensus on the association between cognitive function and mortality, and whether earlier measures of cognitive abilities confound this relationship. In this study, based on the Community Older Adults Health Survey follow-up cohort, we assessed the longitudinal associations of baseline cognitive function and cognitive changes over 1 year with subsequent all-cause mortality among Chinese older adults people aged 60 and above.

## 2 Materials and methods

### 2.1 Study design and participants

This study was based on the Community Older Adults Health Survey follow-up cohort, an ongoing prospective cohort targeting older adults residents in Chinese communities, specifically in Dalang Town, Dongguan City. Initiated in 2018, the cohort annually assesses individuals aged 60 years and above. Baseline data was gathered from surveys distributed in 2018, 2019, and 2020, with follow-up occurring the subsequent year, and ending at death or study closure (August 8th, 2023). Professional medical personnel conducted physical examinations, while face-to-face questionnaires were administered by trained students This study was approved by the Institutional Ethics Review Committee of the Affiliated Hospital of Guangdong Medical University (YJYS2022159), and written informed consent was obtained from all study participants.

**FIGURE 1:**
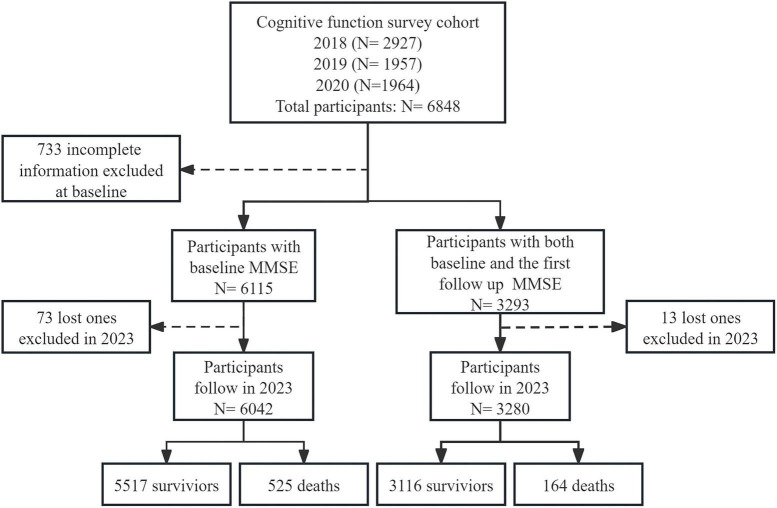
Flow chart of the study population.

A total of 6,848 participants aged 60 and above, without dementia at baseline were included. After excluding 733 individuals with incomplete information and 73 lost to follow-up, 6,042 participants were analyzed for the association between baseline cognitive function and all-cause mortality. A total of 3,293 individuals completed the second survey. After excluding 13 lost to follow-up, 3,280 participants were assessed for the association between cognitive function changes and all-cause mortality (Figure 1).

### 2.2 Outcomes

The survival status and date of death was queried from the Dongguan City death monitoring system using the resident identity card numbers after completion of baseline survey.

### 2.3 Assessing baseline cognitive function and cognitive changes

Cognitive function was assessed using the Chinese version of the Mini-Mental State Examination (MMSE), which was adapted from the international MMSE (Folstein et al., 1975). This has been validated for reliability and validity within the Chinese population (Zhong et al., 2017; Gao et al., 2022). Assessments were conducted through face-to-face interviews without proxies. The MMSE score ranges from 0 to 30, with higher scores indicating better cognitive function: ≥ 26 was considered normal, 21–25 indicated mild cognitive impairment, and 0–20 denoted moderate to severe cognitive impairment (Perneczky et al., 2006).

Change in cognitive function was assessed using the rate of change in MMSE scores, calculated as [(baseline MMSE score - second survey MMSE score)/interval between the two surveys, years]. We categorized the rate of change of MMSE scores into four groups: cognitive improvement (rate less than zero), stable cognitive function (rate equal to zero), slow decline (rate greater than zero but less than or equal to the median decline), and rapid decline (rate greater than the median decline).

### 2.4 Covariates

We adjusted sociodemographic characteristics, health behaviors, and health status in the model. According to the literature (Lipnicki et al., 2019), these factors are considered potential confounders and were included as covariates. Covariates data was collected through questionaire surveys distributed at baseline. Sociodemographic characteristics included age (grouped), sex (man or woman), educational level (illiterate, primary school, junior high school and above), living situation (living alone, with spouse or partner, with children, with spouse and children, with other relatives or non-relatives), marital status (married, widowed, divorced, or never married), and satisfaction with housing. Health behaviors comprised smoking status (current smoker, former smoker, never smoked), alcohol intake (drinker, past, never), exercise frequency (daily, regularly, occasionally, never), and participation in community activities (mostly, sometimes, never). Health status was measured by body mass index (BMI) (underweight (<18.5), normal (18.5–23.9), overweight (24–27.9), obese ( ≥ 28.0) (Jiao et al., 2020), common chronic diseases (self-reported hypertension, diabetes, heart disease, cerebrovascular disease), and mental state (including subjective difficulty concentrating, sadness, loneliness, memory issues, unwilling to go out, fatigue).

### 2.5 Statistical analysis

Cox proportional hazards models were established to determine the relationships between cognitive function and its changes with mortality. All study participants with missing data on the covariates were removed.

Cognitive function and its changes were modeled both as continuous variables (MMSE score; rate of change in MMSE score) and as categorical variables (normal cognitive function, mild impairment, or moderate to severe impairment; improvement, stable, slow decline, and rapid decline). Several models were performed: adjusting for (1) age and sex; (2) added educational level, living situation, marital status, satisfaction with housing, smoking, and health conditions; (3) for the association of changes in cognitive function and all-cause mortality, baseline MMSE score was additionally adjusted. Data are reported as adjusted hazard ratios (HRs) and 95% confidence intervals (CIs).

Restricted cubic spline analysis was performed to examine the relationships between baseline MMSE scores and the rate of change in MMSE scores with mortality, using the median baseline MMSE score (25 points) and the rate of change in MMSE score (0.0%) as reference points, with knots at the 10th, 50th, and 90th percentiles. Survival curves were used to display the survival rate during the follow-up period for baseline cognitive function and changes in cognitive function.

Subgroup analyses were conducted to assess whether associations varied by age (60–69 years, 70–79 years, ≥80 years), and sex (men/women). Interaction terms between baseline cognitive function, cognitive function changes, and the above variables were added separately to the multivariate model. Further subgroup analysis was conducted to explore the associations of cognitive function changes and all-cause mortality at different levels of baseline cognitive function.

Sensitivity analyses addressed follow-up losses by including those lost to follow-up before the end of the study. Additionally, deaths occurring within 1 year of follow-up were excluded, to mitigate cognitive decline’s potential bias.

A two-tailed *P*-value < 0.05 indicated statistical significance. Analyses were performed using R (version 4.2.2) for restricted cubic spline analyses and subgroup analyses; and SPSS 16.0 (IBM Corp., Inc., Chicago, IL, USA) for other analyses.

## 3 Results

### 3.1 Participant characteristics

The median age of the 6,042 participants at baseline was 70.0 years old. Approximately 39% were men, and 20% were illiterate, and 76% were married. At baseline, 56% (*n* = 3,383) of the participants had cognitive impairment, with 33% classified as mild and 23% as moderate to severe impairment. The overall median rate of change in MMSE scores (25th, 75th percentile) was 0.0 (−2.0, 2.0) points per year, with the normal cognitive function group showing a rate of 0.0 (−1.0, 3.0) points, the mild impairment group −1.0 (−4.0, 2.0) points, and the moderate to severe impairment group 0.0 (−4.0, 0.0) points. During a median follow-up period of 3.08 years, 525 deaths (8.69%) were observed (Supplementary Table 1).

### 3.2 Association between baseline cognitive function and mortality

**FIGURE 2:**
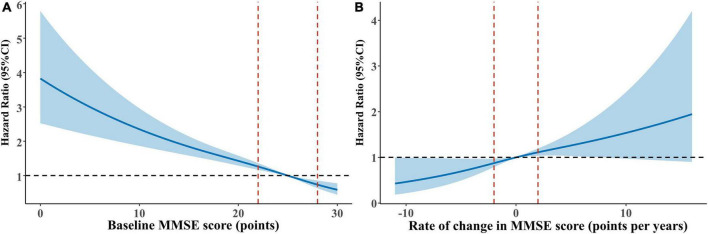
Restricted cubic splines for the association of baseline cognitive function and cognitive function change with all-cause mortality. **(A)** Baseline cognitive function and all-cause mortality: reference point is the median value of baseline MMSE score (25 points). **(B)** Cognitive function change and all-cause mortality: reference point is the median value of cognitive change (0.0%). (Age, sex, education, marital status, living arrangement, housing satisfaction, smoking status, drinking status, physical exercise, community activities, body mass index, hypertension, diabetes, heart disease, cerebrovascular disease, psychological status were adjusted for both panels **(A,B)**. Baseline MMSE score was additionally adjusted for panel **(B)** hazard ratios are indicated by solid lines and 95% confidence intervals by dashed lines, with knots placed at 10th, 50th, and 90th percentiles.

**FIGURE 3:**
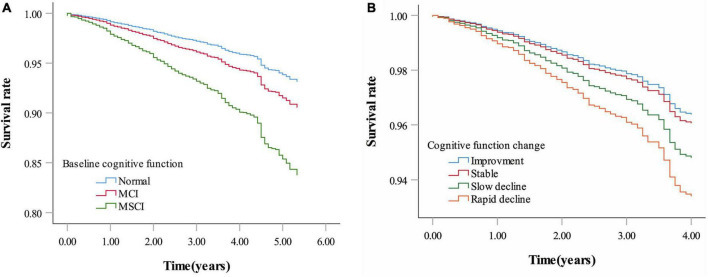
Survival rate by baseline cognitive function and cognitive function change. **(A)** Survival rate by baseline cognitive function. **(B)** Survival rate by cognitive function change. Age, sex, education, marital status, living arrangement, housing satisfaction, smoking status, drinking status, physical exercise, community activities, body mass index, hypertension, diabetes, heart disease, cerebrovascular disease, psychological status were adjusted for both panels **(A,B)**. Baseline MMSE score was additionally adjusted for panel **(B)** (MCI: Mild cognitive impairment; MSCI: Moderate to severe cognitive impairment. Normal: MMSE score ≥ 26 points; MCI: MMSE score 21–25 points; MSCI: MMSE score ≤ 21points. Improvement—rate of change in MMSE score less than zero; stable—rate of change in MMSE score equal to zero; slow decline—rate of change in MMSE score greater than zero but equal to or less than the median of the rate of change showing decline; rapid decline—rate of change in MMSE score greater than the median of the rate of change showing decline).

Over 20,959 person-years of follow-up (normal cognitive function group: 8,867, mild impairment group: 7,135, moderate to severe impairment group: 4,957 person-years), the mortality rate was 2.50 per 100 person-years (with rates of 1.18, 1.93, and 5.69 per 100 person-years for the normal cognitive function, mild impairment, and moderate to severe impairment groups, respectively). Multivariate-adjusted models and restricted cubic splines demonstrated a linear monotonic negative association between baseline cognitive function (as a continuous variable) and all-cause mortality. The risk of death increased by 6% for each one-point decrease in MMSE score (adjusted *HR* = 1.06, 95% *CI*: 1.05–1.08) (Figure 2A and Table 1). Compared with the group with normal cognitive function at baseline, participants in both the mild impairment and moderate to severe impairment groups had an increased risk of all-cause mortality, with greater cognitive impairment associated with higher mortality risk (adjusted *HR* = 1.40, 95% *CI*: 1.07–1.82; *HR* = 2.49, 95% *CI*: 1.91–3.24, respectively) (Figure 3A and Table 1).

**TABLE 1:** The association between baseline cognitive function and all-cause mortality.

Categorical	Participants	Events	Model 1^a^		Model 2^b^	
			HR (95%CI)	*P-*value	HR (95%CI)	*P-*value
MMSE score	6,042	525	1.08 (1.06, 1.09)	<0.001	1.06(1.05, 1.08)	<0.001
Normal	2,659	105	Reference	**–**	Reference	–
MCI	2,012	138	1.53 (1.18, 1.98)	0.001	1.40 (1.07, 1.82)	0.013
MSCI	1,371	282	3.08 (2.39, 3.96)	<0.001	2.49 (1.91, 3.24)	<0.001

HR, hazard ratio; CI, confidence interval; MMSE, Mini-Mental State Examination; MCI, mild cognitive impairment; MSCI, moderate to severe cognitive impairment.

Normal: MMSE score ≥ 26 points; MCI: MMSE score 21–25 points; MSCI: MMSE score ≤ 21points.

a: Adjusted for age and sex.

b: Additionally adjusted for education, marital status, living arrangement, housing satisfaction, smoking status, drinking status, physical exercise, community activities, body mass index, hypertension, diabetes, heart disease, cerebrovascular disease, psychological status.

### 3.3 Association between changes in cognitive function and mortality

Mortality rates for cognitive improvement, stable, slow decline, and rapid decline groups were 1.31, 1.77, 1.61, and 2.35 per 100 person-years (with follow-up durations of 4,038, 2,321, 1,981, and 1,620 person-years, respectively). Multivariate-adjusted models and restricted cubic splines showed a linear monotonic negative association between the rate of cognitive function change and all-cause mortality. The risk of death was 5% higher for each one-point per year decrease in cognitive function change rate (adjusted *HR* = 1.05, 95% *CI*: 1.02–1.08) (Figure 2B and Table 2). Compared with participants with stable cognitive function, those with rapid decline had a 79% increased risk of death (adjusted *HR* = 1.79, 95% *CI*: 1.11–2.87). No significant difference in mortality risk between participants with cognitive improvement or slow decline and those with stable cognitive function (Figure 3B and Table 2).

**TABLE 2:** The association between change in cognitive function and all-cause mortality.

Categorical	Participants	Events	Model 1^a^		Model 2^b^		Model 3^c^	
			HR (95%CI)	*P-*value	HR (95%CI)	*P-*value	HR (95%CI)	*P-*value
Rate of change in MMSE score	3,280	164	1.02 (0.99, 1.06)	0.155	1.02 (0.99, 1.06)	0.180	1.05 (1.02, 1.09)	0.003
Improvement	1,247	53	0.82 (0.54, 1.23)	0.326	0.87 (0.57, 1.31)	0.492	0.93 (0.62, 1.42)	0.748
Stable	725	41	Reference	–	Reference	–	Reference	–
Slow decline	715	32	1.02 (0.54, 1.62)	0.935	1.00 (0.62, 1.62)	0.990	1.46 (0.90, 2.37)	0.129
Rapid decline	593	38	1.15 (0.74, 1.80)	0.531	1.18 (0.74, 1.87)	0.504	1.79 (1.11, 2.87)	0.016

HR, hazard ratio; CI, confidence interval; MMSE, Mini-Mental State Examination. Improvement, rate of change in MMSE score less than zero; stable, rate of change in MMSE score equal to zero; slow decline, rate of change in MMSE score greater than zero but equal to or less than the median of the rate of change in those showing decline; rapid decline, rate of change in MMSE score greater than the median of the rate of change in those showing decline.

a: Adjusted for age and sex.

b: Additionally adjusted for education, marital status, living arrangement, housing satisfaction, smoking status, drinking status, physical exercise, community activities, body mass index, hypertension, diabetes, heart disease, cerebrovascular disease, psychological status.

c: Additionally adjusted for baseline MMSE score.

### 3.4 Subgroup analysis

**FIGURE 4:**
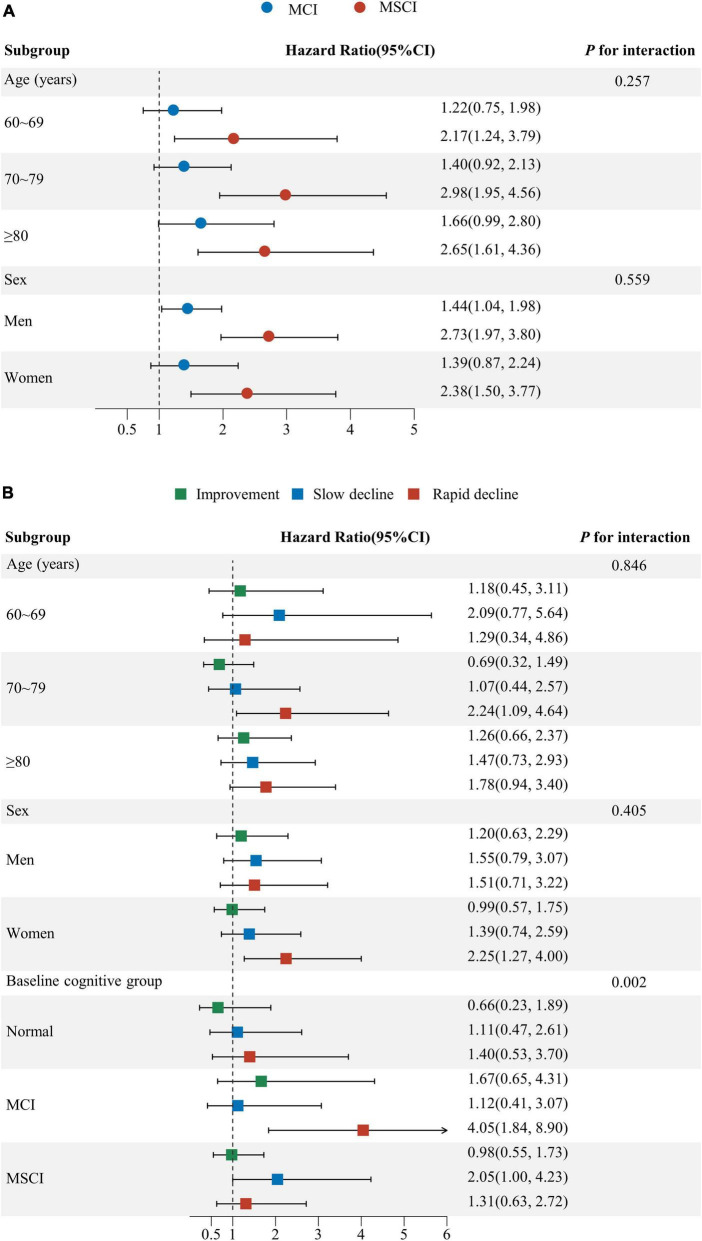
Subgroup analysis for the association of baseline cognitive function and cognitive function change with all-cause mortality. **(A)** Subgroup analysis by age, sex for baseline cognitive function; **(B)** Subgroup analysis by age, sex and baseline cognitive function for cognitive function change. Age, sex, education, marital status, living arrangement, housing satisfaction, smoking status, drinking status, physical exercise, community activities, body mass index, hypertension, diabetes, heart disease, cerebrovascular disease, psychological status were adjusted for Subgroup analysis **(A,B)**. Baseline MMSE score was additionally adjusted for subgroup analysis **(B)** (CI: confidence interval; MCI: mild cognitive impairment; MSCI: moderate to severe cognitive impairment. Normal: MMSE score ≥ 26 points; MCI: MMSE score 21–25 points; MSCI: MMSE score ≤ 21points. Improvement—rate of change in MMSE score less than zero; stable—rate of change in MMSE score equal to zero; slow decline—rate of change in MMSE score greater than zero but equal to or less than the median of the rate of change showing decline; rapid decline—rate of change in MMSE score greater than the median of the rate of change showing decline).

The subgroup analysis showed increased all-cause mortality risk for those with moderate to severe cognitive impairment and rapid cognitive decline. Individuals with moderate to severe impairment, across all age groups, faced a significantly higher mortality risk compared to those with normal cognitive function. Men with mild impairment, and both genders with severe impairment, showed increased mortality risks, more pronounced in men. No significant difference in mortality risk was observed across age and sex subgroups. (*P* for interaction ≥ 0.05) (Figure 4A).

Participants aged 70–79 and women experiencing rapid decline had a higher mortality risk than those with stable function. While rapid decline’s impact on mortality wasn’t significant for normal and severely impaired groups, those with severe impairment and slow decline faced higher mortality compared to those stable. Significant differences in mortality risk due to cognitive function changes were noted across baseline levels (*P* for interaction = 0.002), not by age or sex (*P* for interaction ≥ 0.05) (Figure 4B).

### 3.5 Sensitivity analysis

The results of the sensitivity analysis were similar to those of the main analysis, indicating robust findings when participants lost to follow-up before the end of the study were considered as censored or when participants who died within 1 year of follow-up were excluded (Supplementary Table 2).

## 4 Discussion

In this community-based prospective cohort study, we discovered a significant association between baseline cognitive function and all-cause mortality among individuals aged 60 years and above. First, we observed that the risk of death was significantly higher in older adults with mild and moderate to severe cognitive impairment at baseline, compared with those with normal baseline cognitive function. Moreover, when compared with stable cognitive function, we observed that rapid cognitive decline was associated with a higher all-cause mortality risk. Subgroup analysis confirmed the universality of these relationships among older adults of different age groups, sexes, and baseline cognitive states. We also observed significant differences in the association between cognitive function changes and all-cause mortality across different baseline levels of cognitive function. Compared to participants with normal cognitive function at baseline, individuals who had mild cognitive impairment at baseline and showed a rapid decline in cognitive function, as well as those with moderate to severe cognitive impairment at baseline but experienced a slower decline in cognitive function, both face a higher risk of all-cause mortality.

Our study results are consistent with some existing research. Lower baseline cognitive function is associated with an increased risk of all-cause mortality among older adults individuals. For instance, research conducted on Chinese community-dwelling older adults indicated that a one-point increase in MMSE score correlates with a 4% reduction in mortality risk (Su et al., 2021). Another study highlighted that individuals aged 65 and above with cognitive impairment had more than double the risk of death compared to those with normal cognitive function (Wang et al., 2020). Similar trends were observed in the Bambui Aging Cohort and Korean cohorts, where cognitive impairment was linked with a significantly higher mortality risk (Lee et al., 2018; Diniz et al., 2022). European studies further confirmed that poor cognitive ability is independently associated with increased mortality (Hayat et al., 2018). The mechanisms linking cognitive impairment with all-cause mortality are not entirely clear, but one possible explanation is that individuals with cognitive deficits may have difficulty recognizing and reporting their symptoms and signs, leading to delayed and insufficient diagnosis of health conditions. Additionally, impaired individuals may face challenges in treatment adherence, such as failing to follow medical instructions for medication and inappropriate dietary intake, and often lack physical activity, thereby increasing their future risk of death (Mossakowska et al., 2014).

Regarding changes in cognitive function, current studies suggest that rapid cognitive decline is associated with a higher risk of death (Rajan et al., 2014; Yaffe et al., 2016; Lv et al., 2019). The Chinese Longitudinal Healthy Longevity Survey (CLHLS) indicated that rapid cognitive decline was associated with a 75% higher mortality rate (Lv et al., 2019). Another longitudinal cohort study found that each unit decline in cognitive ability increased the risk of death by 90% (Rajan et al., 2014). Additionally, a cohort study of community-dwelling older women indicated that participants with the fastest rate of cognitive decline (the worst fifth, declining more than 0.14 percentage points per year) had a 28% increased risk of death (Yaffe et al., 2016). This suggests that rapid cognitive decline may be a marker of impending end of life. The relationship between cognitive decline and increased all-cause mortality risk can be explained through various biological and psychosocial mechanisms. Firstly, cognitive decline may be an early marker of neurodegenerative diseases such as Alzheimer’s disease, which are themselves associated with higher mortality rates (Rajan et al., 2014). Research also suggests that psychosocial factors such as loneliness and lack of social support may be linked with accelerated cognitive decline and subsequent increased risk of death. These social factors can exacerbate cognitive decline by increasing stress responses and psychological stress (Ma et al., 2022). Additionally, vascular risk factors, vascular diseases, and mental health status have been reported to be associated with cognitive decline (Lipnicki et al., 2019; Ma et al., 2021a; Lu et al., 2022), but our study found no significant change after adjusting for these variables. Studies also suggest that inflammatory central nervous system diseases, cerebrovascular diseases, toxic-metabolic encephalopathies, and central nervous system tumors can cause rapid cognitive decline (Hermann and Zerr, 2022), suggesting that these factors might concurrently lead to cognitive decline and death, or that cognitive decline, once initiated, may accelerate the dying process. These suggestions warrant further investigation.

In subgroup analysis, we observed the impact of age on the association between cognitive function and all-cause mortality. Adults aged 70–79 years with rapid cognitive decline had a higher risk of all-cause mortality, consistent with previous research (Schupf et al., 2005; Wu et al., 2014). Cognitive decline in relatively young older adults may reflect processes directly related to an increased risk of death, such as the rate of biological aging rather than chronological aging (Bassuk et al., 2000). The association between cognitive function and all-cause mortality was not significant in individuals aged 80 and above, possibly indicating survival bias. We also found that participants with mild cognitive impairment at baseline who experienced rapid cognitive decline, as well as those with moderate to severe cognitive impairment at baseline but experienced a slower decline in cognitive function, both face a higher risk of all-cause mortality. This finding highlights the urgency of early identification and intervention in cognitive impairments, as well as the importance of monitoring cognitive function changes to reduce the risk of mortality. Future work should focus on seeking effective interventions and how to tailor these interventions for different populations (such as subgroups divided by age, gender, and baseline health status). Additionally, exploring the potential biomarkers and mechanisms between cognitive decline and frailty and mortality risk will provide us with a deeper understanding.

This study had several strengths, including the community-based prospective cohort of older adults and it was the first to assess the association between cognitive function and its changes with all-cause mortality from both continuous and categorical perspectives. The findings highlighted the close association of cognitive function and its changes with all-cause mortality among older adults. Given the dynamic nature of older adults’ cognitive function, early screening and identification of cognitive decline to formulate appropriate prevention strategies to halt or delay cognitive decline are crucial (Clare et al., 2017), and this provides a basis for further research.

The study’s insights are limited by its geographical focus on Dongguan City, China, potentially affecting the generalizability to wider populations. The observational spans—4 years for mortality and 3 for cognitive changes—may not fully reveal long-term outcomes. Diagnosing dementia through cognitive assessments may overlook undiagnosed cases, risking misclassification bias. Participant exclusions and unmeasured confounders like diet and inflammation could bias results, necessitating caution. Thus, broader, long-term studies are needed to validate our findings.

## 5 Conclusion

Among Chinese community-dwelling older adults individuals aged 60 years and above, mild and moderate to severe cognitive impairment, as well as rapid cognitive decline, were associated with a higher risk of all-cause mortality. Furthermore, the baseline cognitive function status affected the association between changes in cognitive function and all-cause mortality. Future efforts should focus on regular cognitive assessments of older adults, monitoring disease progression and mortality, and developing preventive strategies to halt or delay the process of cognitive decline and reduce the associated mortality risk.

## Data availability statement

The raw data supporting the conclusions of this article will be made available by the authors, without undue reservation.

## Author contributions

SJL: Conceptualization, Data curation, Formal analysis, Writing – original draft, Writing – review and editing. XH: Investigation, Writing – review and editing. LW: Investigation, Writing – review and editing. XT: Investigation, Writing – review and editing. YO: Investigation, Writing – review and editing. WJ: Investigation, Writing – review and editing. YY: Investigation, Writing – review and editing. JY: Investigation, Writing – review and editing. KC: Investigation, Writing – review and editing. XiZ: Investigation, Writing – review and editing. XuZ: Investigation, Writing – review and editing. JX: Investigation, Writing – review and editing. SBL: Investigation, Writing – review and editing. MY: Conceptualization, Supervision, Writing – review and editing. JN: Conceptualization, Supervision, Writing – review and editing. CP: Writing – review and editing, Validation. XC: Writing – review and editing, Validation.
