# Supplementary material for: The association of cognitive function and its changes with all-cause mortality among community-dwelling older adults
Source: Front Aging Neurosci. 2024 Jun 12;16:1419235. doi: 10.3389/fnagi.2024.1419235 (PMC11199401; doi:10.3389/fnagi.2024.1419235)
Supplement: Supplementary file 1 [file Table_1.DOCX]

***Supplementary Materials***

**1 Supplementary Figures and Tables**

**1.1 Supplementary Table 1 Characteristics of 6,042 participants at baseline**

| Variables | Total  (n=6,042) | Normal  (n=2,659) | MCI  (n=2,012) | MSCI  (n=1,371) | *P value* |
| --- | --- | --- | --- | --- | --- |
| Age, years^*^ | 70.0 [66.0, 76.0] | 69.0 [66.0, 72.0] | 70.0 [66.0, 75.0] | 77.0 [70.0, 83.0] | <0.001 |
| Age group |  |  |  |  | <0.001 |
| 60~69 years | 2,563 (42.42) | 1,380 (51.90) | 881 (43.79) | 302 (22.03) |  |
| 70~79years | 2,532 (41.91) | 1,142 (42.95) | 869 (43.19) | 521 (38.00) |  |
| ≥80 years | 947 (15.67) | 137 (5.15) | 262 (13.02) | 548 (39.97) |  |
| Sex |  |  |  |  | <0.001 |
| Men | 2,382 (39.42) | 1,381 (51.94) | 656 (32.60) | 345 (25.16) |  |
| Women | 3,660 (60.58) | 1,278 (48.06) | 1,356 (67.40) | 1,026 (74.84) |  |
| Education |  |  |  |  | <0.001 |
| Illiterate | 1,203 (19.91) | 149 (5.60) | 427 (21.22) | 627 (45.73) |  |
| Primary school | 3,499 (57.91) | 1,598 (60.10) | 1,259 (62.57) | 642 (46.83) |  |
| Junior high school and above | 1,340 (22.18) | 912 (34.30) | 326 (16.20) | 102 (7.44) |  |
| Marital status |  |  |  |  | <0.001 |
| Married | 4,616 (76.40) | 2,230 (83.87) | 1,556 (77.34) | 830 (60.54) |  |
| Widowed | 1,398 (23.14) | 411 (15.46) | 450 (22.37) | 537 (39.17) |  |
| Divorced / Unmarried | 28 (0.46) | 18 (0.68) | 6 (0.30) | 4 (0.29) |  |
| Living arrangement |  |  |  |  | <0.001 |
| Alone | 791 (13.09) | 205 (7.71) | 219 (10.88) | 367 (26.77) |  |
| With spouse | 1,736 (28.73) | 777 (29.22) | 584 (29.03) | 375 (27.35) |  |
| With children | 1,464 (24.23) | 567 (21.32) | 566 (28.13) | 331 (24.14) |  |
| With spouse and children | 2,011 (33.28) | 1,103 (41.48) | 632 (31.41) | 276 (20.13) |  |
| With others | 40 (0.66) | 7 (0.26) | 11 (0.55) | 22 (1.60) |  |
| Housing satisfaction |  |  |  |  | <0.001 |
| Satisfied | 1,788 (29.59) | 909 (34.19) | 611 (30.37) | 268 (19.55) |  |
| General | 869 (14.38) | 359 (13.50) | 264 (13.12) | 246 (17.94) |  |
| Unsatisfied | 3,385 (56.02) | 1,391 (52.31) | 1,137 (56.51) | 857 (62.51) |  |
| Smoking status |  |  |  |  | <0.001 |
| Current | 1,208 (19.99) | 688 (25.87) | 360 (17.89) | 160 (11.67) |  |
| Former | 862 (14.27) | 353 (13.28) | 282 (14.02) | 227 (16.56) |  |
| Never | 3,972 (65.74) | 1,618 (60.85) | 1,370 (68.09) | 984 (71.77) |  |
| Drinking status |  |  |  |  | <0.001 |
| Current / Former | 554 (9.17) | 341 (12.82) | 147 (7.31) | 66 (4.81) |  |
| Never | 5,488 (90.83) | 2,318 (87.18) | 1,865 (92.69) | 1,305 (95.19) |  |
| Physical exercise |  |  |  |  | <0.001 |
| Everyday | 4,319 (71.48) | 2,053 (77.21) | 1,460 (72.56) | 806 (58.79) |  |
| <5 times/week | 234 (3.87) | 103 (3.87) | 77 (3.83) | 54 (3.94) |  |
| <2 times/week | 253 (4.19) | 93 (3.50) | 78 (3.88) | 82 (5.98) |  |
| Never | 1,236 (20.46) | 410 (15.42) | 397 (19.73) | 429 (31.29) |  |
| Community activities |  |  |  |  | <0.001 |
| Always | 1,775 (29.38) | 760 (28.58) | 627 (31.16) | 388 (28.30) |  |
| Occasionally | 482 (7.98) | 278 (10.46) | 154 (7.65) | 50 (3.65) |  |
| Never | 3,785 (62.64) | 1,621 (60.96) | 1,231 (61.18) | 933 (68.05) |  |
| Body mass index |  |  |  |  | <0.001 |
| Underweight(<18.5) | 371 (6.14) | 122 (4.59) | 121 (6.01) | 128 (9.34) |  |
| Normal(18.5~23.9) | 2,743 (45.40) | 1,131 (42.53) | 932 (46.32) | 680 (49.60) |  |
| Overweight(24.0~27.9) | 2,078 (34.39) | 1,015 (38.17) | 683 (33.95) | 380 (27.72) |  |
| Obese(>28.0) | 850 (14.07) | 391 (14.70) | 276 (13.72) | 183 (13.35) |  |
| Hypertension | 2,439 (40.37) | 1,022 (38.44) | 820 (40.76) | 597 (43.54) | 0.007 |
| Diabetes | 584 (9.67) | 249 (9.36) | 211 (10.49) | 124 (9.04) | 0.296 |
| Heart disease | 260 (4.30) | 111 (4.17) | 78 (3.88) | 71 (5.18) | 0.170 |
| Cerebrovascular disease | 89 (1.47) | 32 (1.20) | 24 (1.19) | 33 (2.41) | 0.005 |
| Psychological status |  |  |  |  |  |
| Feel pretty worthless | 510 (8.44) | 151 (5.68) | 173 (8.60) | 186 (13.57) | <0.001 |
| Feel sad / depressed | 1,017 (16.83) | 323 (12.15) | 322 (16.00) | 372 (27.13) | <0.001 |
| Feel lonely | 494 (8.18) | 120 (4.51) | 145 (7.21) | 229 (16.70) | <0.001 |
| Forgetfulness | 1,877 (31.07) | 762 (28.66) | 603 (29.97) | 512 (37.35) | <0.001 |
| Prefer stay at home | 461 (7.63) | 142 (5.34) | 158 (7.85) | 161 (11.74) | <0.001 |
| Feel tired | 630 (10.43) | 219 (8.24) | 212 (10.54) | 199 (14.51) | <0.001 |
| Baseline MMSE score, points^*^ | 25 [21, 28] | 28 [27, 29] | 24 [22, 25] | 17 [14, 19] | - |
| Final MMSE score, points^*#^ | 25 [22, 29] | 28 [25, 29] | 25[22, 27] | 17 [14, 23] | - |
| Rate of change in MMSE score, points/year^*$^ | 0.0 [-2.0, 2.0] | 0.0 [-1.0, 3.0] | -1.0 [-4.0, 2.0] | 0.0 [-4.0, 0.0] | <0.001 |
| Vital status |  |  |  |  | <0.001 |
| Survived | 5,517 (91.31) | 2,554 (96.05) | 1,874 (93.14) | 1,089 (79.43) |  |
| Deceased | 525 (8.69) | 105 (3.95) | 138 (6.86) | 282 (20.57) |  |
| Duration of follow-up, years^*^ | 3.08 [2.83, 4.25] | 3.00 [2.83, 4.25] | 3.08 [2.83, 4.33] | 4.17 [2.92, 4.46] | <0.001 |

Abbreviation: *MCI,* mild cognitive impairment; *MSCI,* moderate to severe cognitive impairment; *MMSE* Mini-Mental State Examination

Normal: MMSE score ≥ 26 points; MCI: MMSE score 21–25 points; MSCI: MMSE score ≤ 21points

*Data are expressed as the median [25th percentile, 75th percentile]

^#^A total of 3,280 participants completed the final MMSE score examination at 1 year after baseline measurement. ^$^Rate of change in MMSE score - (baseline MMSE score - final MMSE score)/the interval between two examinations (years), calculated with the data from the 3,280 participants who completed two examinations.

**1.2 Supplementary Table 2 Sensitivity analysis for the association between baseline cognitive function plus cognitive function change and all-cause mortality**

| Variables | Categorical | Participants | Events | HR(95%CI) | *P* value |
| --- | --- | --- | --- | --- | --- |
| Sensitivity analysis 1: Participants lost to follow-up were treated as censored at the end of the study | | | | | |
| Baseline MMSE score | - | 6,115 | 525 | 1.06(1.05, 1.08) | <0.001 |
| Baseline cognitive function | Normal | 2,690 | 105 | Reference | - |
|  | MCI | 2,037 | 138 | 1.40(1.07, 1.82) | 0.013 |
|  | MSCI | 1,388 | 262 | 2.49(1.91, 3.25) | <0.001 |
| Rate of change in MMSE | - | 3,293 | 164 | 1.05(1.02, 1.08) | 0.002 |
| Cognitive function change | Improvement | 1,251 | 53 | 0.92(0.60, 1.40) | 0.690 |
|  | Stable | 727 | 41 | Reference | - |
|  | Slow decline | 717 | 32 | 1.33(0.81, 2.18) | 0.261 |
|  | Rapid decline | 598 | 38 | 1.70(1.04, 2.77) | 0.034 |
| Sensitivity analysis 2: Exclusion of deaths that occurred in a year of follow-up | | | | | |
| Baseline MMSE score | - | 5,933 | 416 | 1.05(1.04, 1.08) | <0.001 |
| Baseline cognitive function | Normal | 2,639 | 85 | Reference | - |
|  | MCI | 1,985 | 111 | 1.36(1.01, 1.82) | 0.04 |
|  | MSCI | 1,309 | 220 | 2.31(1.72, 3.11) | <0.001 |
| Rate of change in MMSE | - | 3,266 | 150 | 1.05(1.02, 1.09) | 0.003 |
| Cognitive function change | Improvement | 1,241 | 47 | 0.86(0.56, 1.32) | 0.487 |
|  | Stable | 724 | 40 | Reference | - |
|  | Slow decline | 711 | 28 | 1.34(0.81, 2.22) | 0.261 |
|  | Rapid decline | 590 | 35 | 1.70(1.04, 2.77) | 0.033 |

Abbreviations: *HR,* hazard ratio, *CI,* confidence interval; *MMSE,* Mini-Mental State Examination; *MCI,* Mild cognitive impairment; *MSCI,* moderate to severe cognitive impairment

Normal: MMSE score ≥ 26 points; MCI: MMSE score 21–25 points; MSCI: MMSE score ≤ 21points

Improvement, rate of change in MMSE score less than zero; stable, rate of change in MMSE score equal to zero; slow decline, rate of change in MMSE score greater than zero but equal to or less than the median of the rate of change in those showing decline; rapid decline, rate of change in MMSE score greater than the median of the rate of change in those showing decline

Sensitivity analyses 1 and 2: Age, sex, education, marital status, living arrangement, housing satisfaction, smoking status, drinking status, physical exercise, community activities, body mass index, hypertension, diabetes, heart disease, cerebrovascular disease, and psychological status were adjusted for baseline MMSE score and baseline cognitive function, and baseline MMSE score was additionally adjusted for rate of change in MMSE and cognitive function change.
